# An exploration strategy improves the diversity of de novo ligands using deep reinforcement learning: a case for the adenosine A_2A_ receptor

**DOI:** 10.1186/s13321-019-0355-6

**Published:** 2019-05-24

**Authors:** Xuhan Liu, Kai Ye, Herman W. T. van Vlijmen, Adriaan P. IJzerman, Gerard J. P. van Westen

**Affiliations:** 1 Drug Discovery and Safety, Leiden Academic Centre for Drug Research, Einsteinweg 55, Leiden, The Netherlands; 2 Omics and Omics Informatics, Xi’an Jiaotong University, 28 Xianning W Rd, Xi’an, China; 3 Janssen Pharmaceutica NV, Turnhoutseweg 30, 2340 Beerse, Belgium

**Keywords:** Deep learning, Adenosine receptors, Cheminformatics, Reinforcement learning, Exploration strategy

## Abstract

Over the last 5 years deep learning has progressed tremendously in both image recognition and natural language processing. Now it is increasingly applied to other data rich fields. In drug discovery, recurrent neural networks (RNNs) have been shown to be an effective method to generate novel chemical structures in the form of SMILES. However, ligands generated by current methods have so far provided relatively low diversity and do not fully cover the whole chemical space occupied by known ligands. Here, we propose a new method (DrugEx) to discover de novo drug-like molecules. DrugEx is an RNN model (generator) trained through reinforcement learning which was integrated with a special exploration strategy. As a case study we applied our method to design ligands against the adenosine A_2A_ receptor. From ChEMBL data, a machine learning model (predictor) was created to predict whether generated molecules are active or not. Based on this predictor as the reward function, the generator was trained by reinforcement learning without any further data. We then compared the performance of our method with two previously published methods, REINVENT and ORGANIC. We found that candidate molecules our model designed, and predicted to be active, had a larger chemical diversity and better covered the chemical space of known ligands compared to the state-of-the-art.

**Electronic supplementary material:**

The online version of this article (10.1186/s13321-019-0355-6) contains supplementary material, which is available to authorized users.

## Introduction

G Protein-Coupled Receptors (GPCRs) are the largest family of cell membrane-bound proteins [1], containing more than 800 members encoded by approximately 4% of human genes. GPCRs are central to a large number of essential biological processes, including cell proliferation, cell survival, and cell motility [2]. Currently, GPCRs form the main target of approximately 34% of all FDA approved drugs [3, 4]. One of the most extensively studied GPCRs is the human adenosine A_2A_ receptor (A_2A_R), which has been shown to be a promising drug target for among others Parkinson’s disease, cardiovascular diseases, and inflammatory disorders [5]. Multiple crystal structures with different ligands have been resolved [6, 7], and data on the biological activity of thousands of chemical compounds against the receptor was made available in the public ChEMBL database [8]. Considering the amount of data available and our in-house expertise we exploited machine learning methods to design novel ligands with predicted activity on the A_2A_R.

Over the last years, deep learning (DL) has been at the forefront of great breakthroughs in the field of artificial intelligence and its performance even surpassed human abilities for image recognition and natural language processing [9]. Since then, deep learning is gradually being applied to other data rich fields [10, 11]. In drug discovery DL has been used to construct quantitative structure-activity relationship (QSAR) models [12] to predict the properties of chemical compounds, such as toxicity, partition coefficient and affinity for specific targets, etc [13, 14]. Most commonly pre-defined descriptors such as Extended Connectivity Fingerprint (ECFP) [15] were used as input to construct fully-connected neural networks [16]. More recently studies were published using other methods wherein neural networks extract the descriptor from chemical structures automatically and directly, such as Mol2Vec [17], DruGAN [18], GraphConv [19], etc.

In addition to these *prediction* applications, DL can also be used in chemical structure *generation* [14]. Gupta et al. [20] constructed a recurrent neural network (RNN) model to learn the syntax of the SMILES notation and generate novel SMILES representing novel molecules. In addition, Olivecrona et al. [21] combined RNNs and reinforcement learning (RL) to generate SMILES formatted molecules that are enriched for chemical and biological properties (named REINVENT). RL has been instrumental in the construction of “AlphaGo” designed by DeepMind, which defeated one of the best human Go players [22]. Finally, similar to generative adversarial networks (GANs) for generating images [23], Benjamin et al. exploited the GAN for a sequence generation model [24] to generate molecules with multi-objective reinforcement learning (named ORGANIC) [25].

In order to maximize the chance to find interesting hits for a given target, generated drug candidates should (a) be chemically diverse, (b) possess biological activity, and (c) contain similar (physico) chemical properties to already known ligands [26]. Although several groups have studied the application of DL for generating molecules as drug candidates, most current generative models cannot satisfy all of these three conditions simultaneously [27]. Considering the variance in structure and function of GPCRs and the huge space of drug candidates, it is impossible to enumerate all possible virtual molecules in advance [28]. Here we aimed to discover de novo drug-like molecules active against the A_2A_R by our proposed new method DrugEx in which an exploration strategy was integrated into a RL model. The integration of this function ensured that our model generated candidate molecules similar to known ligands of the A_2A_R with great chemical diversity and predicted affinity for the A_2A_R. All python code for this study is freely available at http://github.com/XuhanLiu/DrugEx.

## Dataset and methods

### Data source

Drug-like molecules were collected from the ZINC database (version 15) [29]. We randomly chose approximately one million SMILES formatted molecules that met the following criteria: − 2 <predicted logP < 6 and 200 < molecular weight (MW) < 600. The dataset (named *ZINC* hereafter) finally contained 1,018,517 molecules and was used for SMILES syntax learning. Furthermore, we extracted the known ligands for the A_2A_R (ChEMBL identifier: CHEMBL251) from ChEMBL (version 23) [30]. If multiple measurements for the same ligand existed, the average pCHEMBL value (pKi or pIC50 value) was calculated and duplicate items were removed. If the pCHEMBL value was < 6.5 or the compound was annotated as “Not Active” it was regarded as a negative sample; otherwise, it was regarded as a positive sample. In the end this dataset (named as *A2AR*) contained 2420 positive samples and 2562 negative samples.

### Prediction model (QSAR)

Binary classification through QSAR modelling was used as prediction task. Input data for the model were ECFP6 fingerprints with 4096 bits calculated by the RDKit Morgan Fingerprint algorithm with a three-bond radius [31]. Hence, each molecule in the dataset was transformed into a 4096D vector. Model output value was the probability whether a given chemical compound was active based on this vector. Four algorithms were benchmarked for model construction, Random Forest (RF), Support Vector Machine (SVM), Naïve Bayesian (NB), and deep neural network (DNN). The RF, SVM and NB models were implemented through Scikit-Learn [32], and DNN through PyTorch [33]. In RF, the number of trees was set as 1000 and split criterion was “*gini*”. In SVM, a radial basis function (RBF) kernel was used and the parameter space of *C* and *γ* were set as [2^−5^, 2^15^] and [2^−15^, 2^5^], respectively. In DNN, the architecture contained three hidden layers activated by rectified linear unit (ReLU) between input and output layers (activated by sigmoid function), the number of neurons were 4096, 8000, 4000, 2000 and 1 for each layer. With 100 epochs of training process 20% of hidden neurons were randomly dropped out between each layer. The binary cross entropy was used to construct the loss function and optimized by Adam [34] with a learning rate of 10^−3^. The area under the curve (AUC) of the receiver operator characteristic (ROC) curves was calculated to compare their mutual performance.

### Generative model

Starting from the SMILES format, each molecule in the *ZINC* set was split into a series of tokens, standing for different types of atoms, bonds, and grammar controlling tokens. Then, all tokens existing in this dataset were collected to construct the SMILES vocabulary. The final vocabulary contained 56 tokens (Additional file 1: Table S1) which were selected and arranged sequentially into valid SMILES sequence following the correct grammar.

**Fig. 1:**
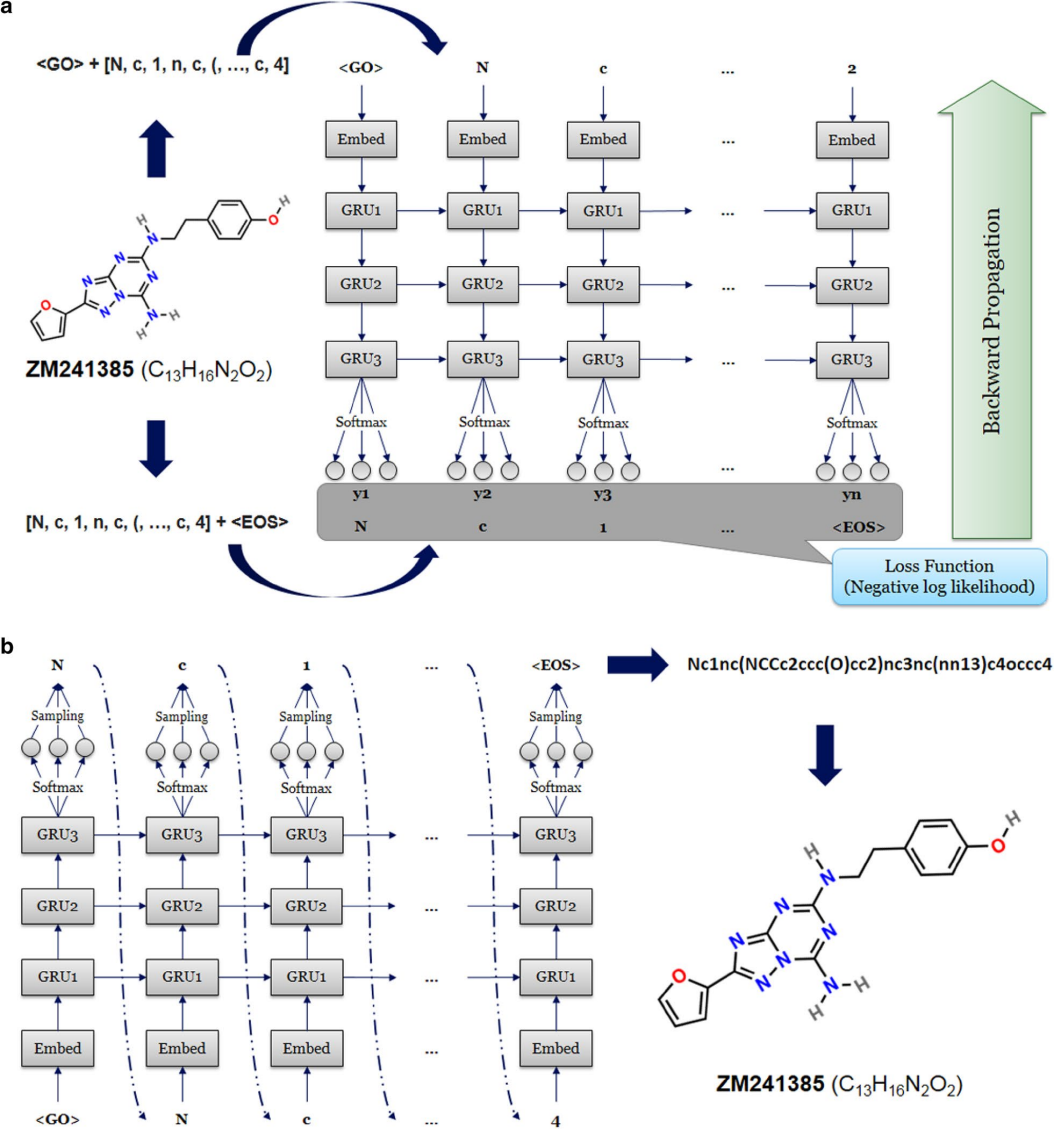
Architecture of recurrent neural networks for the training and sampling processes with A_2A_R antagonist ZM241385 as an example. **a** In the training process of RNNs, each molecule is decomposed to a series of tokens and then taken as input. Subsequently, the input and output are combined with a start token and an end token, respectively. **b** Beginning with the start token “GO”, the model calculates the probability distribution of each token in the vocabulary. For each step, one of the available tokens is randomly chosen based on the probability distribution and is again received by RNNs as input to calculate the new probability distribution for the next step. The maximum of steps was set as 100 and the process will end if the end token “EOS” is sampled or the maximum of steps is reached

The RNN model constructed for sequence generation contained six layers: one input layer, one embedding layer, three recurrent layers and one output layer (Fig. 1). After being represented by a sequence of tokens, molecules can be received as categorical features by the input layer. In the embedding layer, vocabulary size, and embedding dimension were set to 56 and 128, meaning each token could be transformed into a 128d vector. For the recurrent layer, a gated recurrent unit (GRU) [35] was used as the recurrent cell with 512 hidden neurons. The output at each position was the probability that determined which token in the vocabulary would be chosen to construct the SMILES string.

During the training process we put the start token at the beginning of a batch of data as input and the end token at the end of the same batch of data as output. This ensures that the generative network could choose correct tokens based on the sequence it had generated (Fig. 1a). A negative log likelihood function was used to construct the loss function to guarantee that the token in the output sequence had the largest probability to be chosen after being trained. In order to optimize the parameters of the model, the Adam algorithm [34] was used for optimization of loss function. Here, the learning rate was set at 10^−3^, batch size was 500, and training steps set at 1000 epochs.

### Reinforcement learning

**Fig. 2:**
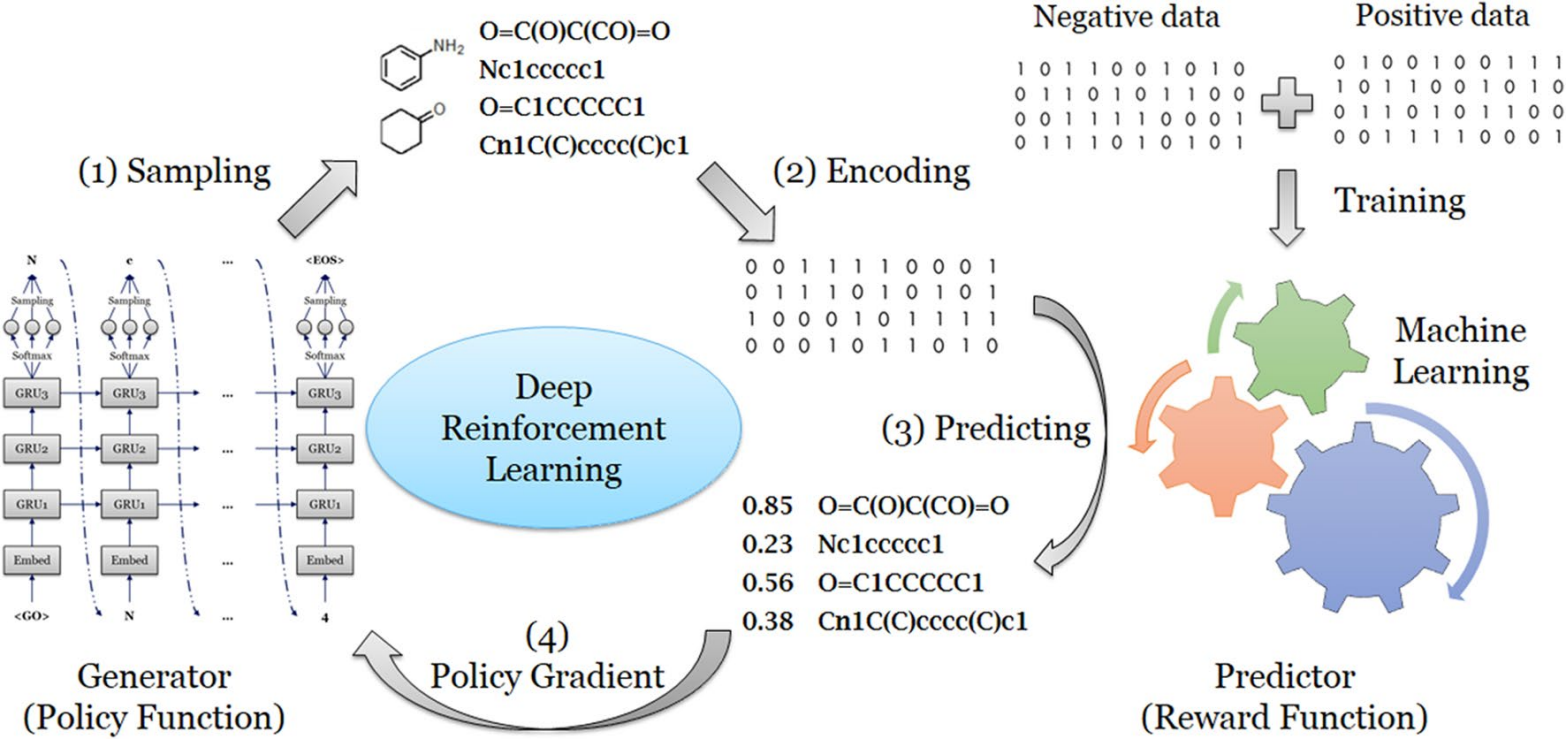
The workflow of deep reinforcement learning. For each loop, it contains several steps: (1) a batch of SMILES sequences was sampled by the RNN generator. (2) Each generated molecule represented by this SMILES format was encoded into a fingerprint; (3) a probability score of activity on the A_2A_R was assigned to each molecule, calculated by the QSAR model which had been trained in advance. (4) All of the generated molecules and their scores were sent back for training of the generator with the policy gradient method

SMILES sequence construction under the RL framework can be viewed as a series of decision-making steps (Fig. 2). At each step, the model determines the optimal token from the vocabulary based on the generated sequence in previous steps. However, the pure RNN model cannot guarantee that the percentage of desired molecules (i.e. predicted to be biologically active on the A_2A_R) being generated is as large as possible. To solve this problem RL is an appropriate method as it increases the probability of those molecules with higher rewards and avoids generating those molecules with lower rewards. We regarded the generator as the policy function and the predictor as the reward function. The generator *G*_*θ*_ was updated by employing a policy gradient based on the expected end reward received from the predictor *Q*. The objective function could be designated as generating a sequence from the start state to maximize the expected end reward [24].

$$ J\left( \theta \right) = E\left[ {R(y_{1:T} ) |\theta } \right] = \mathop \sum \limits_{t = 1}^{T} log G_{\theta } \left( {y_{t} |y_{1:t - 1} } \right) \cdot \left( {Q\left( {y_{1:T} } \right) - \beta } \right) $$

Here *R* is the reward for a complete sequence which is given by the prediction model *Q*; the generative model *G*_*θ*_ can be regarded as policy function to determine the probability of each token from the vocabulary to be chosen. The parameter *β* was the baseline of the reward, meaning that if the reward score was not larger than the baseline, the model would take it as a minus score or punishment. The goal of the generative model is to construct a sequence which can obtain the highest score as judged by the predictor.

### Exploration strategy

**Fig. 3:**
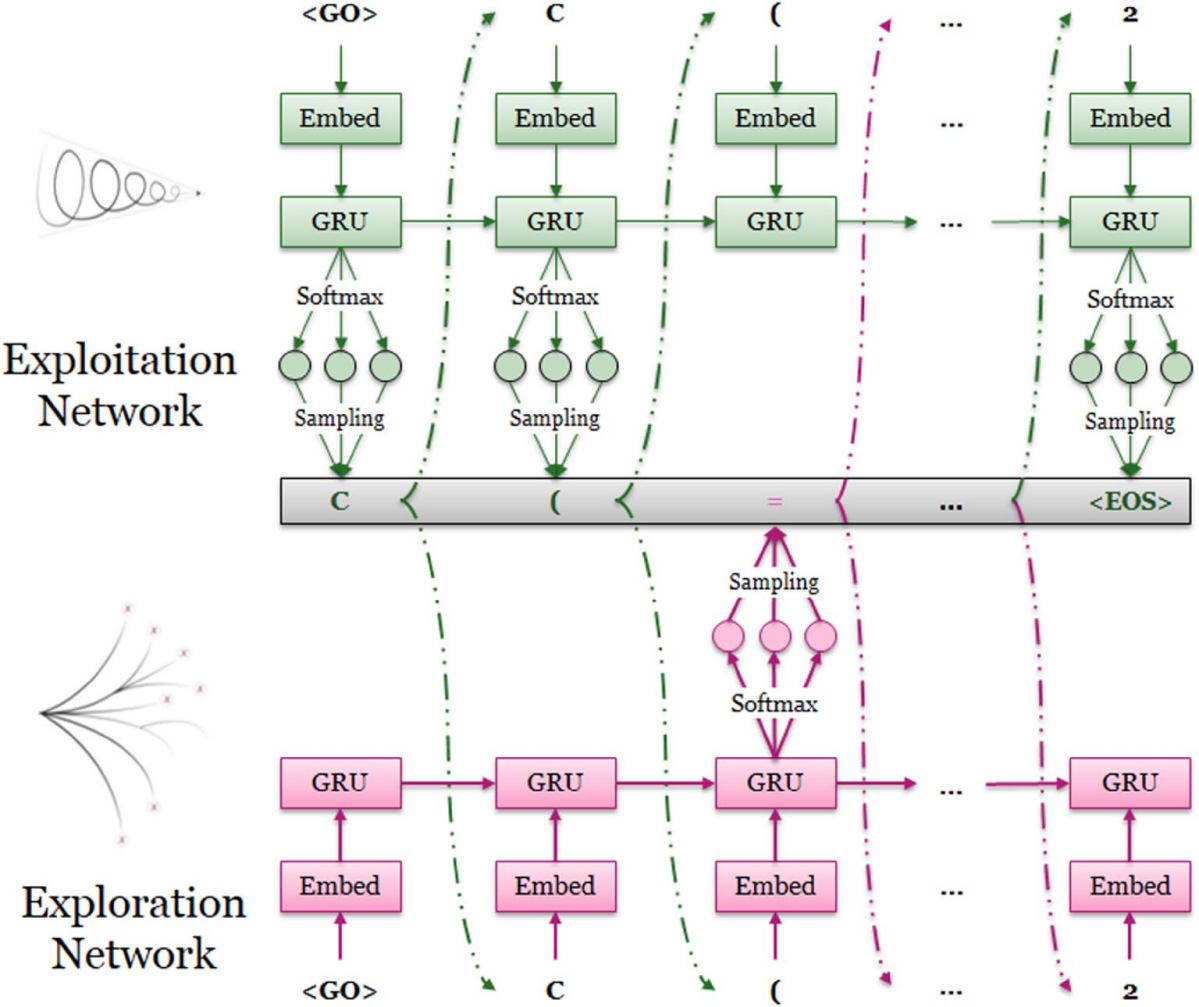
Molecule generation with the assistance of the exploration strategy during the training process. For each step of token selection, a random variable was generated between 0 and 1. If the value is larger than a pre-set threshold (exploring rate, *ε*), the probability distribution is determined by the current generator (exploitation network, *G*_*θ*_). Otherwise, it was determined by the exploration network (*G*_*φ*_)

In order to improve the diversity of generated molecules, the token selection was not only determined by the generator constructed by the RNN model as described above, but also by a second fixed well-trained RNN model (Fig. 3). The RNN requiring training is deemed the ‘exploitation network’ (*G*_*θ*_) and the fixed RNN (not requiring training) is deemed the ‘exploration network’ (*G*_*φ*_). Both had an identical network architecture. We define “exploring rate” (*ε*) in the range (0.0, 1.0) to determine which fraction of steps was determined by the exploration network. During the training process, each SMILES sequence was generated through the collaboration of these two RNNs. At each step a random number in [0.0, 1.0] was generated. If the value was smaller than *ε*, the *G*_*φ*_ would determine which token to be chosen, and vice versa. After the training process was finished, we removed *G*_*φ*_ and only *G*_*θ*_ was left as the final model of DrugEx for molecule generation.

### Molecular diversity

The Tanimoto-similarity was used for measuring the similarity of molecules. Given two compounds *a* and *b* and their ECFP6 fingerprints *m*_*a*_ and *m*_*b*_, the Tanimoto-similarity is defined as:

$$ T_{s} \left( {a, b} \right) = \frac{{\left| {m_{a} \cap m_{b} } \right|}}{{\left| {m_{a} \cup m_{b} } \right|}} $$

where |*m*_*a*_ ⋂ *m*_*b*_| represents the number of common fingerprint bits, and | *m*_*a*_ ∪ *m*_*b*_ | donates the total number of fingerprint bits. The Tanimoto-distance is defined as:

$$ T_{d} \left( {a, b} \right) = 1 - T_{s} \left( {a, b} \right) $$

Similar to Benhenda [27], the diversity I of a set of molecules A (with size of |A|) is defined as the average of the Tanimoto-distance of every pair of molecules:

$$ I\left( A \right) = \frac{1}{{\left| A \right|^{2} }}\mathop \sum \limits_{{\left( {a, b} \right) \in A \times A}} T_{d} \left( {a, b} \right) $$

In a given set of molecules, the less similar each two molecules are, the larger the value of its diversity will be.

## Results and discussion

### Performance of predictors

**Fig. 4:**
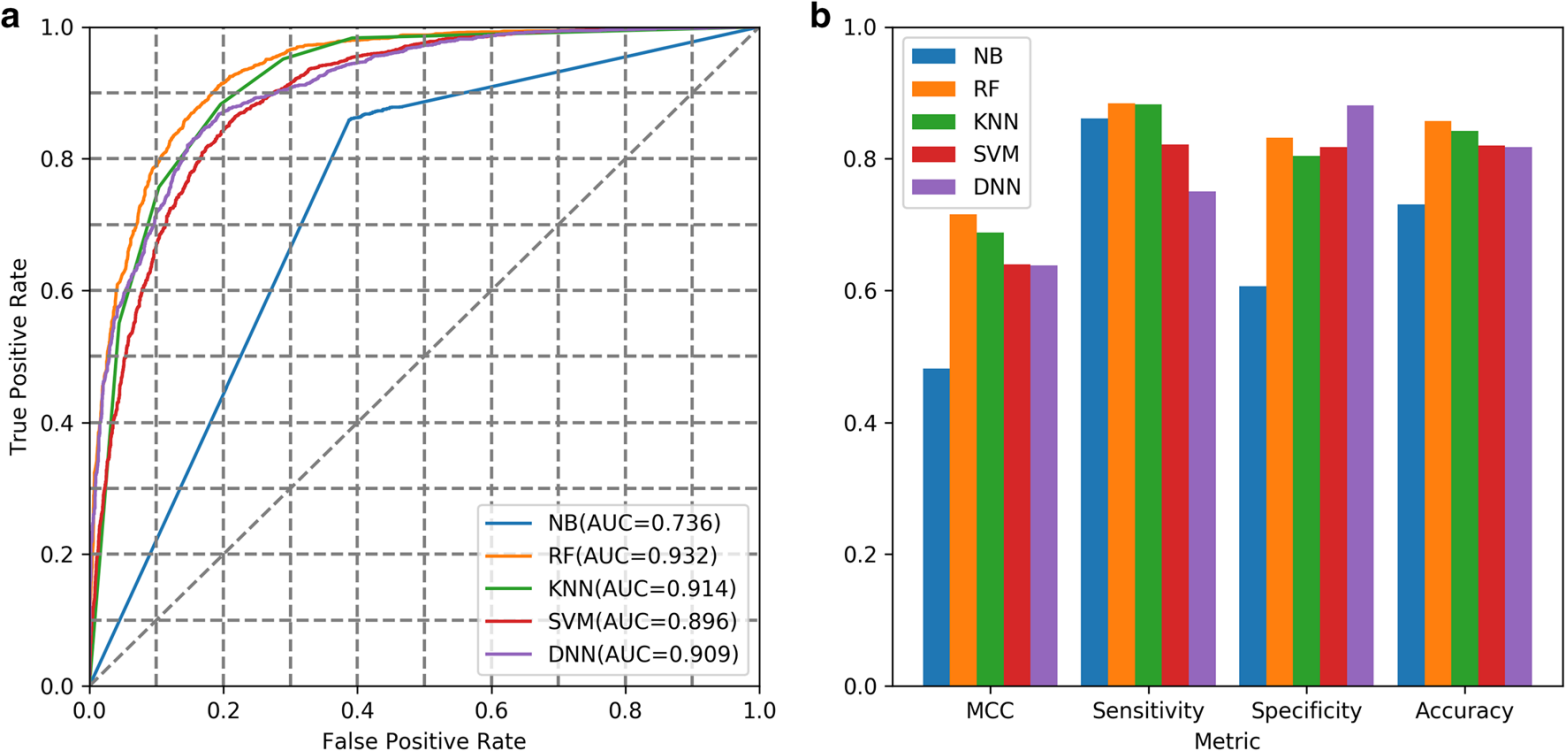
Performance of five different machine learning models based on fivefold cross validation in the *A2AR* set with different metrics, including AUC of ROC curve (**a**), MCC, Sensitivity, Specificity and Accuracy values (**b**). Except for specificity, the RF achieved highest scores among these models based on such measurements

All molecules in the *A2AR* set were used for training the QSAR models, after being transformed into ECFP6 fingerprints. We then tested the performance of these different algorithms with fivefold cross validation of which the ROC curves are shown in Fig. 4. The RF model achieved the highest value of AUC, Matthews correlation coefficient (MCC), Sensitivity, and Accuracy, despite its Specificity being slightly lower than DNN. Hence this model was chosen as our predictor whose output would be regarded as the reward for the generator in RL. In our previous study [16], the performance of the DNN was better than that of the RF on the chemical space of the whole ChEMBL database. A possible reason for the difference observed here can be that both the size of the *A2AR* set and its chemical diversity were much smaller than that of the ChEMBL set. This could have a negative influence on DNN, which had more parameters to be optimized than RF. Selecting the predictor was a critical step in this study, as this model would be used to determine whether the following generated molecules were active or inactive.

### SMILES libraries generation

**Fig. 5:**
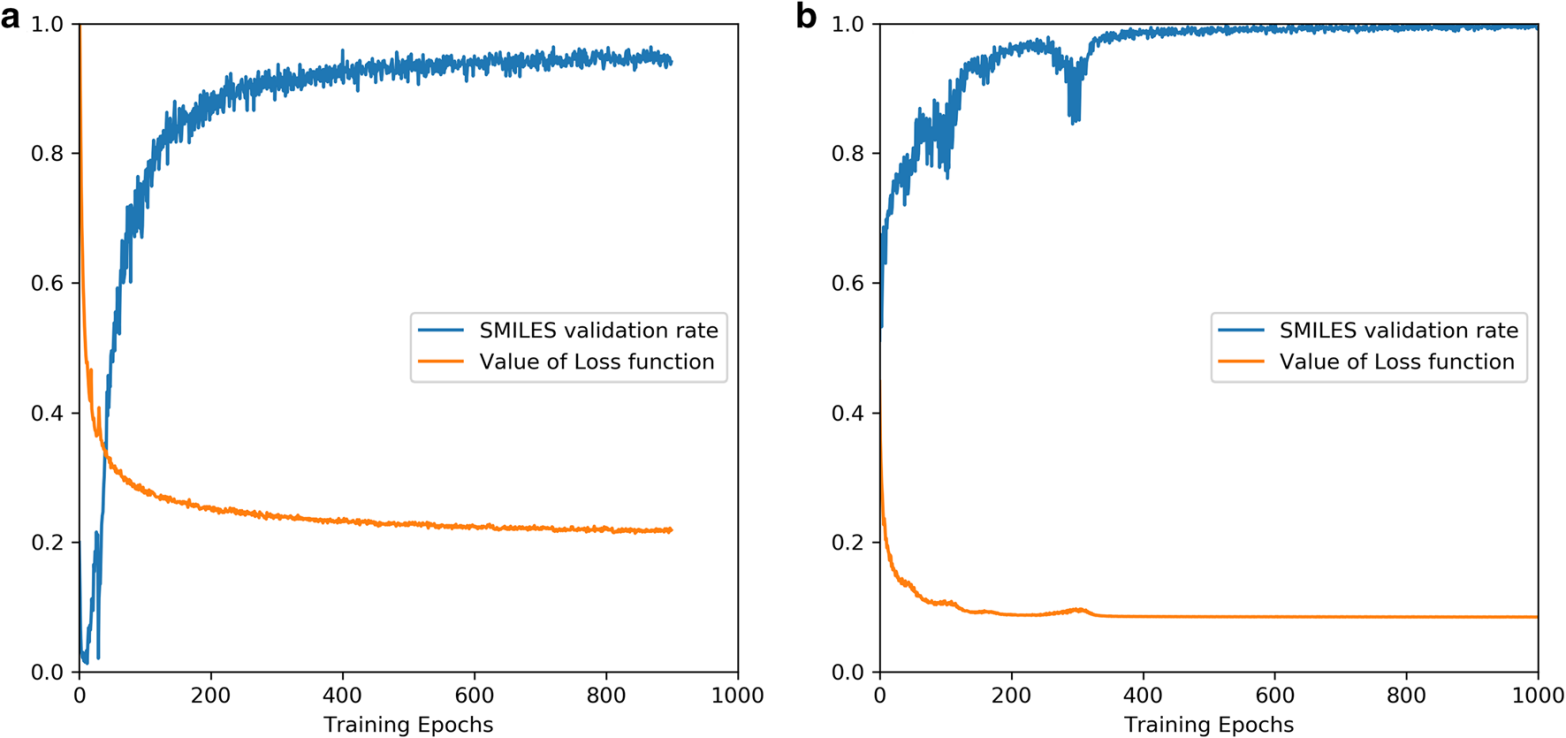
The value of the loss function and the percentage of valid SMILES sequences during the pre-training process on the *ZINC* set (**a**) and fine-tuning process on the *A2AR* set (**b**). The model was well pre-trained after 300 epochs and these two values converged to 0.19 and 93.88%, respectively. The performance of the fine-tuned model converged after 400 epochs with the two values reaching 0.09 and 99.73%, respectively

For the training of RNNs all molecules in the *ZINC* set were used as training set after being decomposed into the tokens which belonged to our vocabulary set. Here, we defined that a SMILES sequence was valid if it could be parsed by RDKit [31]. During the training process, the percentage of valid SMILES sequences through 1000 times sampling was calculated and was then recorded with the value of the loss function at each epoch (Fig. 5a). After about 300 epochs, the loss function had converged, indicating the model was trained well.

**Fig. 6:**
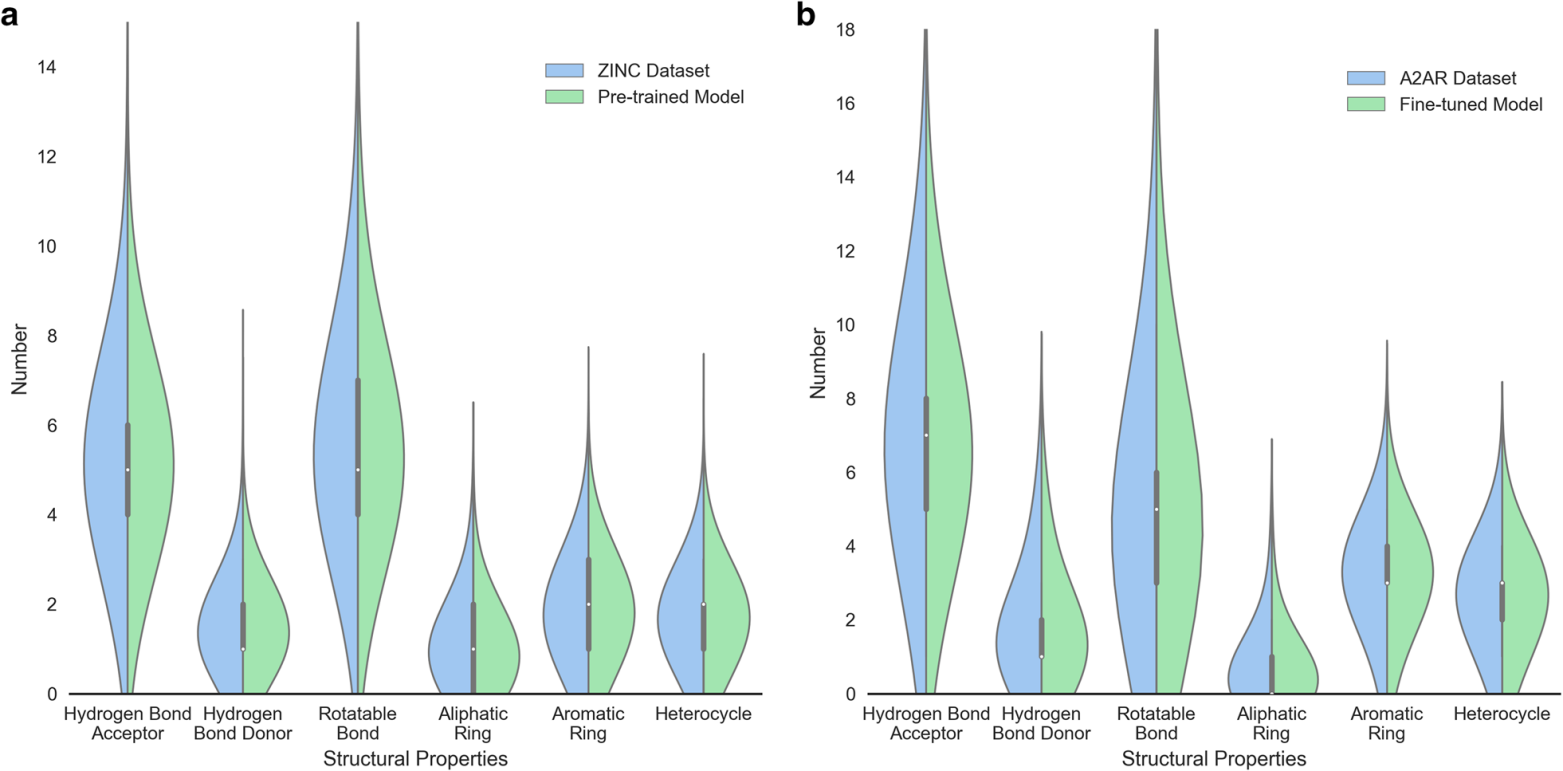
Comparison of the properties of generated molecules by the pre-trained (**a**) and fine-tuned models (**b**) and molecules in the *ZINC* set (**a**) and the *A2AR* set (**b**), respectively. These properties included the number of hydrogen bond acceptors/donors, rotatable bonds, aliphatic rings, aromatic rings, and heterocycles

**Fig. 7:**
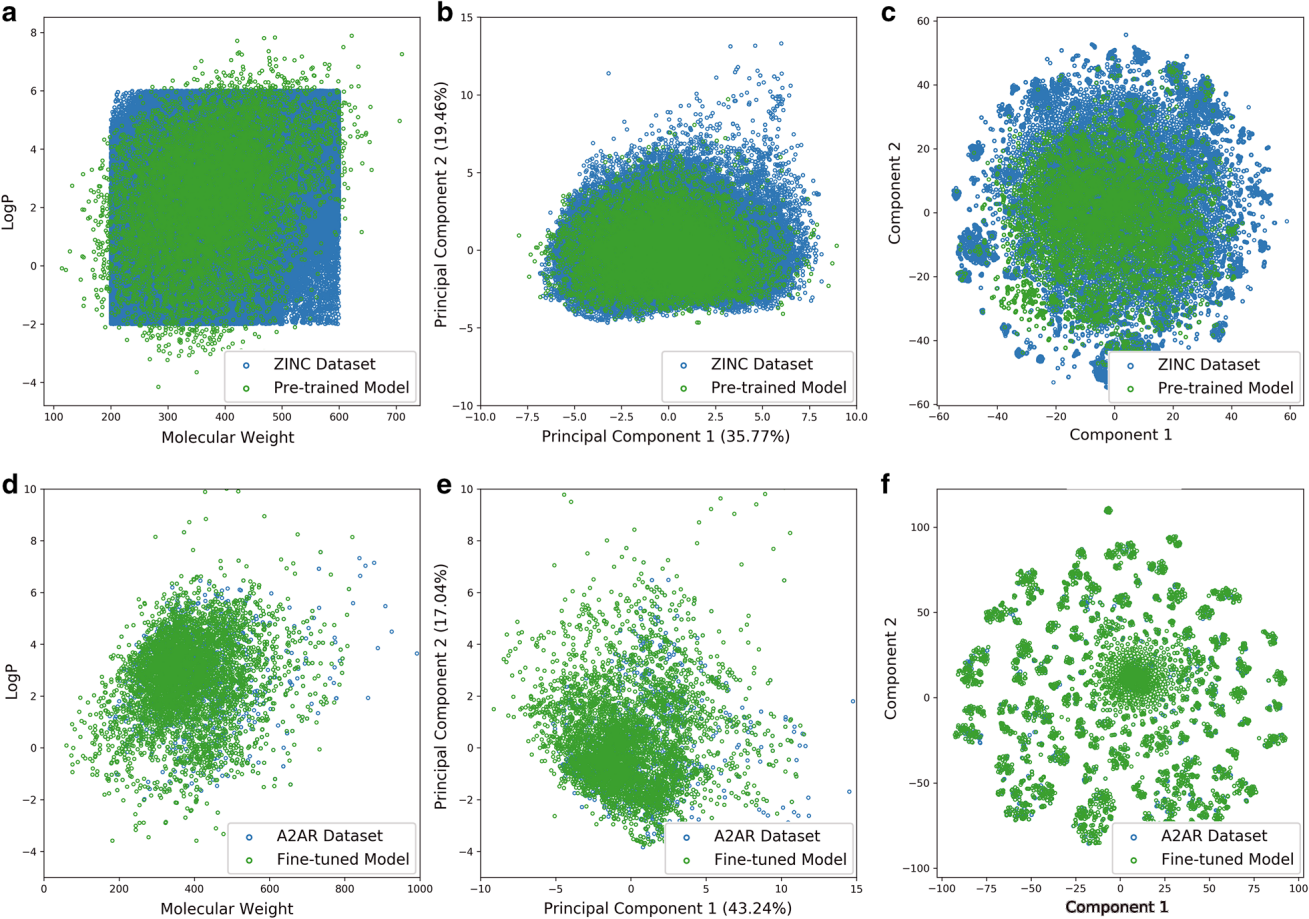
The chemical space of generated molecules by the pre-trained model with the *ZINC* set (**a**–**c**) and the fine-tuned model with the *A2AR* set (**d**–**f**). The chemical space was represented by either logP ~ MW (**a**, **d**), first two components in PCA on PhysChem descriptors (**c**, **e**), and t-SNE on ECFP6 fingerprints (**d**, **f**)

Subsequently, we sampled 10,000 SMILES sequences based on this well-trained model and found that 93.88% of these sequences were grammatically correct SMILES. We then compared some properties of these generated molecules with those in the training set, including number of hydrogen bond donors/acceptors, rotatable bonds, and different kind of ring systems (Fig. 6a). The distribution of these properties in the generated molecules highly resembles the molecules in the *ZINC* set. The logP ~ MW plot (Fig. 7a) shows that most generated molecules were drug-like molecules and cover the vast majority of the square space occupied by the *ZINC* set. Besides these eight properties, we also calculated 11 other physicochemical properties (including topological polar surface area, molar refractivity, the fraction of sp^3^ hybridized carbon atoms and number of amide bonds, bridgehead atoms, heteroatoms, heavy atoms, spiroatoms, rings, saturated rings, valence electrons) to form a 19D physicochemical descriptors (PhysChem). Subsequently, principal component analysis (PCA) and t-distributed stochastic neighbor embedding (t-SNE) [36, 37] were employed for dimensionality reduction and chemical space visualization with the PhysChem and ECFP6 descriptors of these molecules, respectively. Generated molecules were found to cover almost the whole region occupied by molecules in the *ZINC* set (Fig. 7b, c) although the number of these generated molecules was less than 1% of the number of molecules in the *ZINC* set.

Subsequently we used the *A2AR* set to fine-tune this pre-trained model with 1000 epochs (Fig. 5b). After sampling another 10,000 times, we performed the same comparison with the *A2AR* set with respect to the properties mentioned above (Fig. 6b) and investigated the chemical space represented by logP ~ MW (Fig. 7d), the first two components of the PCA on PhysChem descriptors (Fig. 7e) and the t-SNE on ECFP6 fingerprints (Fig. 7f), yielding results similar to the model without fine-tuning but then focused on the *A2AR* chemical space. These results prove that RNN is an appropriate method to learn the SMILES grammar and to construct molecules similar to the ligands in the training set, which has also been shown in other work [20, 38].

### Conditional SMILES generation

The RNN model trained on the *ZINC* set was used as an initial state for the policy gradient in RL. After the training process of RL and the model converged, 10,000 SMILES sequences were generated for performance evaluation. However, after removal of duplicates in these sequences, only less than 10 unique molecules were left which were similar to compounds in the *A2AR* set. When checking the log file of the training process and we noticed that these duplicated sequences were frequently sampled at each epoch and its duplication rate increased gradually. In order to decrease the bias caused by these molecules with high frequency, we removed all duplicated sequences sampled at each epoch for training with the policy gradient. We found that subsequently almost all of the molecules generated according to this procedure were located outside of the drug-like region with regard to logP ~ MW plot (Additional file 1: Figure S2). This problem might be caused by the bias of the predictor. ECFP is a substructure-based fingerprint, implying that if the molecule contains some critical substructures, it will be prone to be predicted as active. That was the reason why generated SMILES sequences contained a large number of repetitive motifs. Several research groups have made improvements to guarantee that the final model has ability to generate drug-like candidate molecules [21, 25]. In the next section, we will describe our proposed method, “DrugEx” by integrating an exploration strategy to solve this problem and compare it to existing methods.

### Exploration strategy

**Fig. 8:**
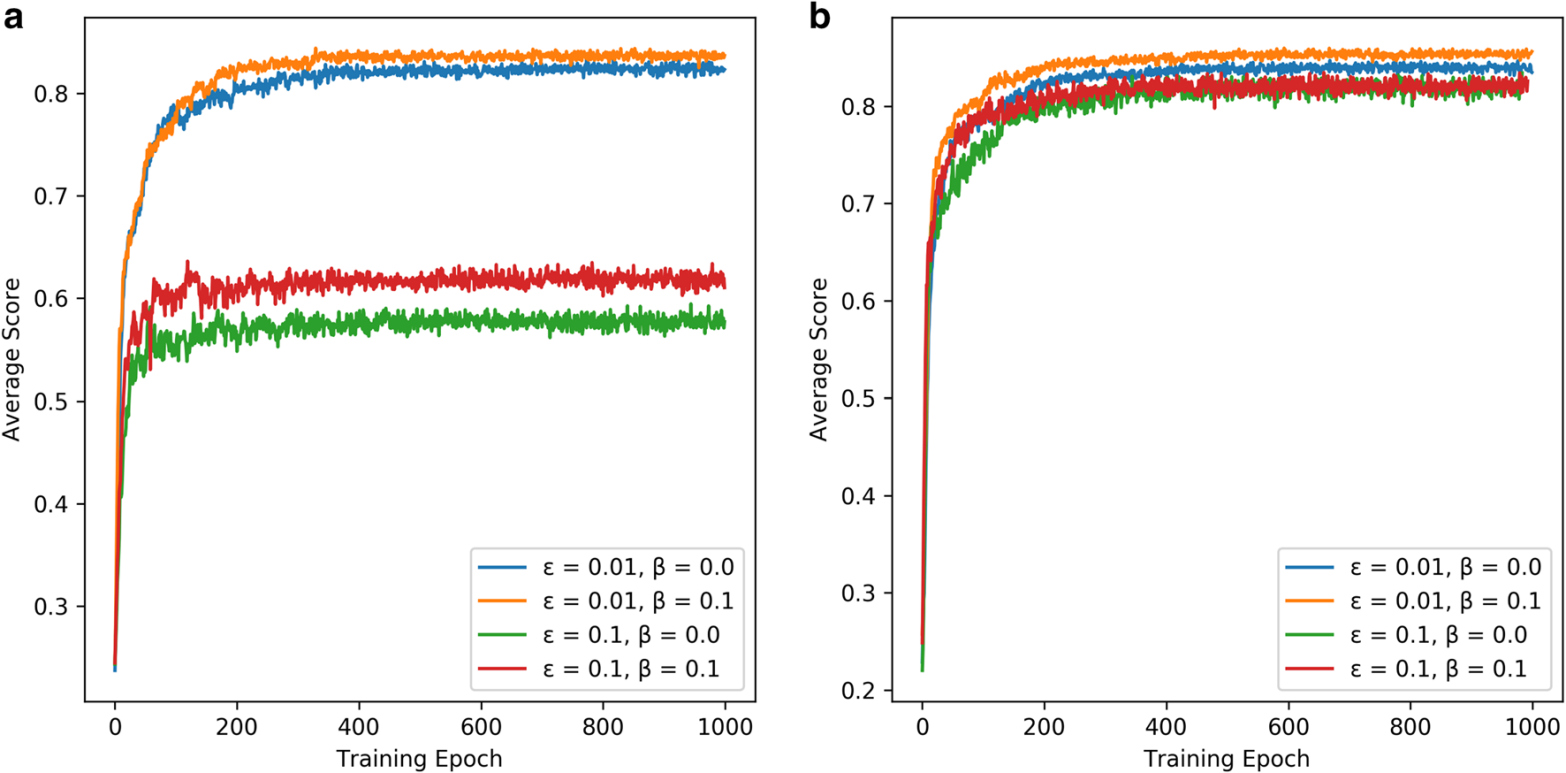
The average score of generated SMILES sequences during the training processes of deep reinforcement learning with different *ε*, *β and G*_*φ*_. The pre-trained model on the *ZINC* set (**a**) and the fine-tuned model on the *A2AR* set (**b**) were used as *G*_*φ*_. After 200 epochs, the average scores for all training processes converged and whole of these models were well trained

**Fig. 9:**
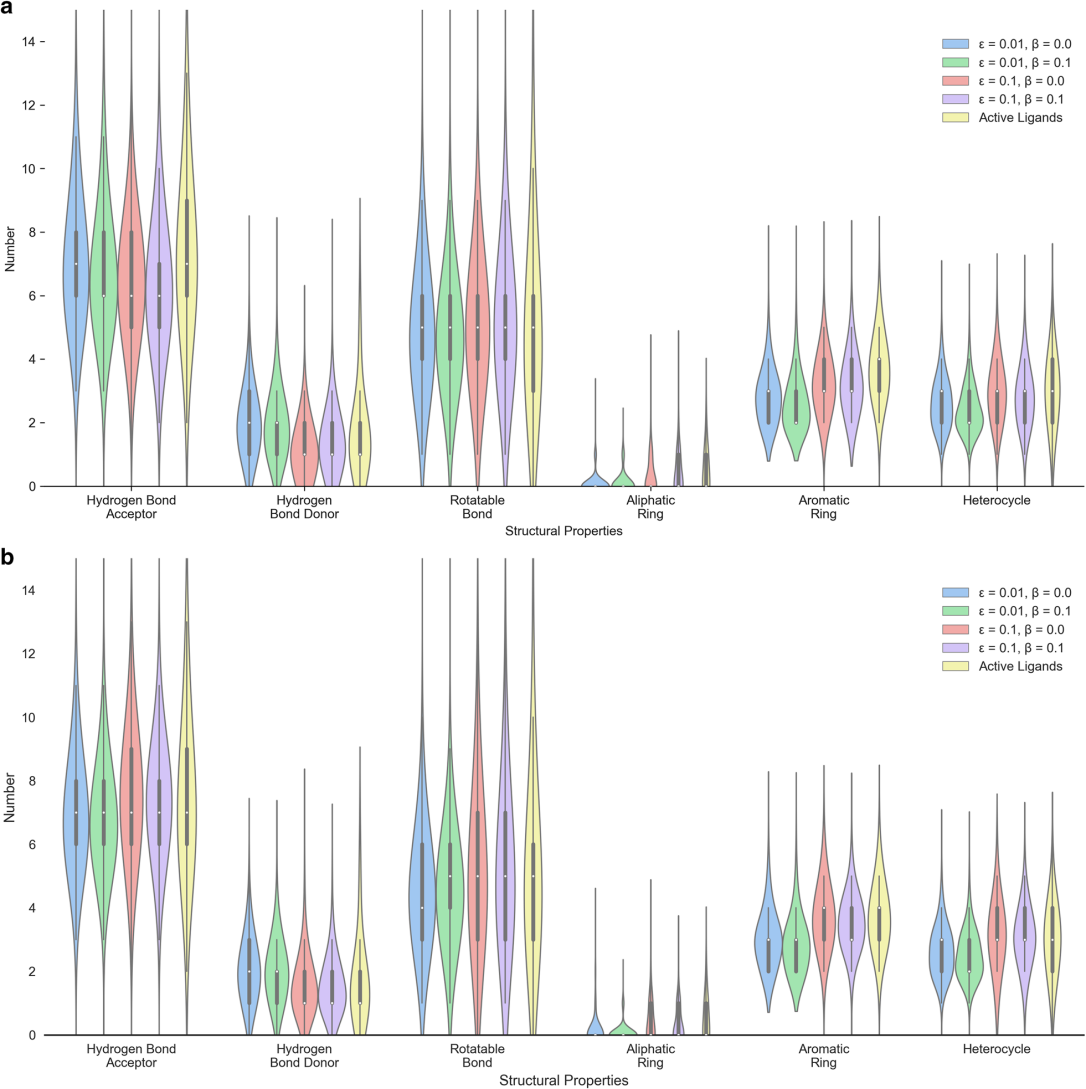
Comparison of the properties of generated molecules by RL models with different *ε*, *β* and *G*_*φ*_. The pre-trained model on the *ZINC* set (**a**) and the fine-tuned model on the *A2AR* set (**b**) were used as *G*_*φ*_. These properties included the number of hydrogen bond donors/acceptors, rotatable bonds, aliphatic rings, aromatic rings, and heterocycles

**Fig. 10:**
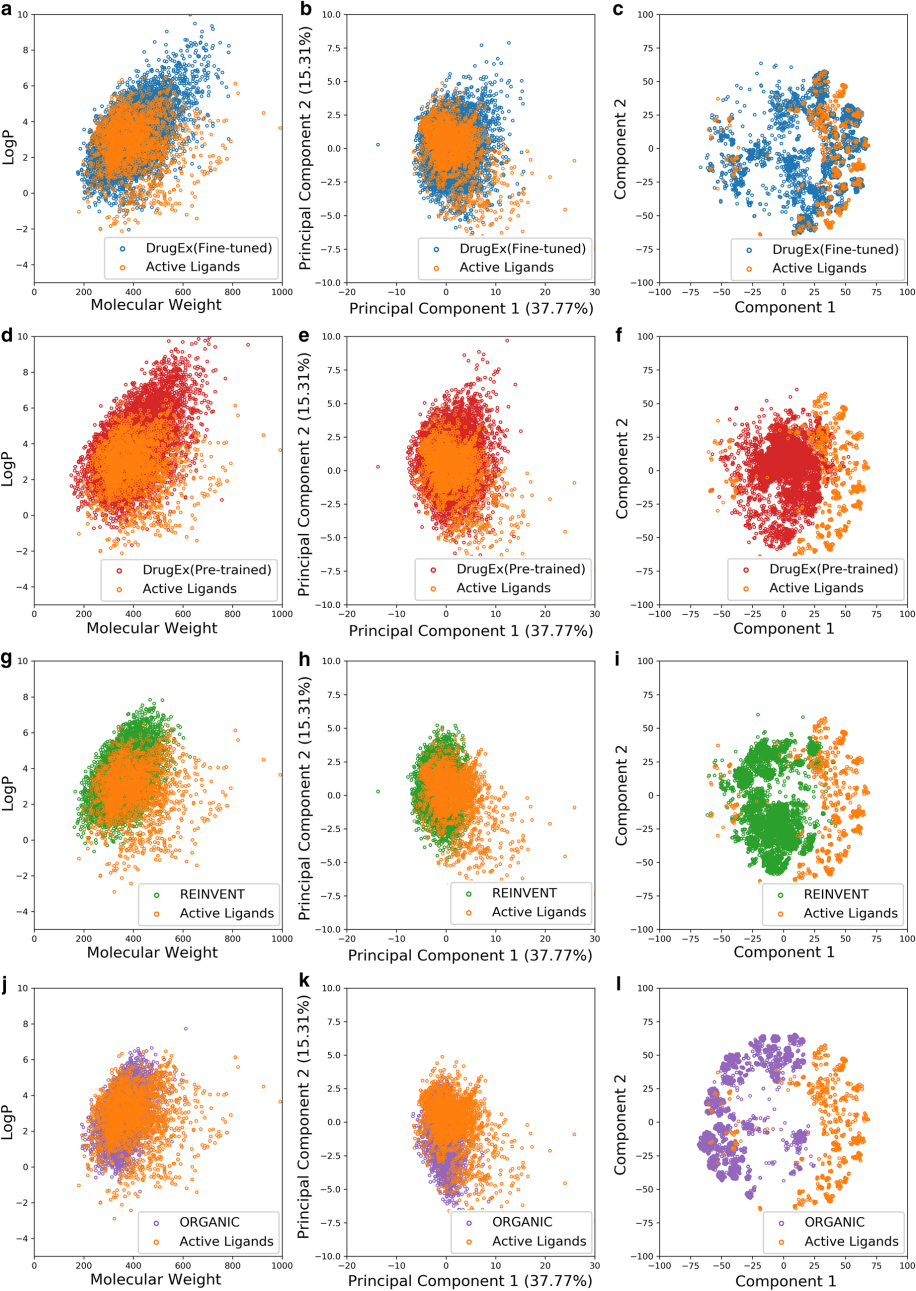
Comparison of the chemical space of active ligands in the *A2AR* set and generated molecules by DrugEx (fine-tuned, **a**–**c**), DrugEx (pre-trained, **d**–**f**), REINVENT (**g**–**i**), and ORGANIC (**j**–**l**). Chemical Space was represented by logP ~ MW (**a**, **d**, **g**, **j**), the first two components in PCA on PhysChem descriptors (**b**, **e**, **h**, **k**), and t-SNE on ECFP6 fingerprints (**c**, **f**, **i**, **l**)

During the training process, the generated sequence is determined by both the *G*_*θ*_ and the *G*_*φ*_ where *ε* determines how many contributions the *G*_*φ*_ made. The *G*_*φ*_ and *G*_*θ*_ were both initialized by the pre-trained RNN model on the *ZINC* set. The *G*_*φ*_ was fixed and only parameters in the *G*_*θ*_ were updated. In order to optimize parameters, the parameter space was designated [0.01, 0.05, 0.10, 0.15, 0.20, 0.25] and [0.0, 0.1] for *ε* and *β*, respectively. After the model converged at 200 epochs (Fig. 8a), the performance of these models was evaluated subsequently based on 10,000 sampled sequences. Firstly, it was found that the number of duplicate SMILES notations was reduced dramatically and almost all SMILES notations represented drug-like molecules (Figs. 9a, 10d). Table 1 shows that when *ε* was increased, the model generated fewer active ligands for the A_2A_R but the diversity of generated molecules (represented as unique desired SMILES) increased significantly. It was also observed that with higher *ε*, the distribution of different kinds of ring systems in the generated desired molecules became more similar to the known active ligands in the *A2AR* set (Fig. 9a). The results with different combination of *ε* and *β* are shown in Additional file 1: Figure S3. Here, *ε* = 0.1 was selected as the optimal exploration rate by considering the combination between diversity and unique desired rate. The *G*_*φ*_ can hence help the model produce more molecules *similar* to known active ligands of the given target but not *identical* to them. At higher *ε*, the baseline can help the model improve the average score and generate more desired molecules. However, this effect was less pronounced at lower values of *ε*. It is worth noticing in this study that if *β* > 0.1 or *ε* > 0.25, the training process of the generative model did not converge.

**Table 1:** Comparison of the performance of the different methods

	DrugEx (Pre-trained)				DrugEx (Fine-tuned)				REINVENT	ORGANIC	Pre-trained	Fine-tuned
*ε*	0.01	0.01	0.1	0.1	0.01	0.01	0.1	0.1	–	–	–	–
*β*	0.0	0.1	0.0	0.1	0.0	0.1	0.0	0.1	–	–	–	–
Valid SMILES	98.3%	98.9%	95.9%	98.8%	99.1%	99.0%	98.2%	97.5%	98.8%	99.8%	93.9%	96.2%
Desired SMILES	97.5%	98.0%	74.6%	80.9%	98.3%	98.5%	94.4%	94.5%	98.2%	99.8%	0.7%	47.9%
Unique SMILES	96.5%	96.3%	73.0%	80.0%	96.5%	96.6%	84.8%	86.0%	95.8%	94.8%	0.7%	22.7%
Diversity	0.74	0.75	0.80	0.80	0.75	0.74	0.80	0.80	0.75	0.67	0.83	0.82

These methods included DrugEx with different *ε*, *β* and *G*_*φ*_ (shown in the parentheses), REINVENT, ORGANIC, the pre-trained network, and the fine-tuned network (both without using DrugEx)

Subsequently, the fine-tuned network was used as *G*_*φ*_ to be involved in our proposed training method of RL. After the training process converged at 200 epochs (Fig. 8b), 10,000 SMILES were generated. Compared to the pre-trained network, there were more unique molecules generated (Table 1), most of which were drug-like compounds (Figs. 9b, 10a). However, with appropriate *ε* the fine-tuned network helped the model generate more valid desired SMILES than with the pre-trained network. At the same time the duplication rate was also increased and there were more repetitive molecules being generated. A possible reason is that the percentage of active ligands was higher in the *A2AR* set than in the *ZINC* set, while the size of the *A2AR* set was much smaller than the *ZINC* set, causing a higher number of duplicated samples generated by the fine-tuned model. In addition, a PCA showed that the fine-tuned network was more effective than the pre-trained network as *G*_*φ*_, as it helped the model in generating molecules with larger chemical diversity while maintaining a higher similarity to the known active ligands (Figs. 9, 10). These results prove that the exploration strategy is an effective way to assist the model training for generating novel molecules with similar chemical and biological properties to existing molecules in a specific part of chemical space.

### Comparison with other methods

Several papers on SMILES generation using deep learning have been published. Olivecrona et al. [21] proposed a method named “REINVENT”, in which a new loss function was introduced based on the Bayesian formula for RL,

$$ L\left( \theta \right) = \left[ {logP_{Prior} \left( {y_{1:T} } \right) + \sigma R\left( {y_{1:T} } \right) - logP_{Agent} \left( {y_{1:T} } \right)} \right]^{2} $$

The authors used all molecules in the ChEMBL database to pre-train an RNN model as the *Priori*. With the parameter *σ*, they integrated the reward *R* of each SMILES into the loss function. The final *Agent* model was regarded as the Posteriori and trained with the policy gradient. Finally, they successfully identified a large number of active ligands against the dopamine D2 receptor (DRD2).

Likewise, Benjamin et al. [25] proposed another method named “ORGANIC” by combining a GAN model for sequence generation and a prediction model to form a comprehensive reward function for RL.

$$ R\left( {y_{1:t} } \right) = \lambda R_{d} \left( {y_{1:T} } \right) + \left( {1 - \lambda } \right)R_{c} \left( {y_{1:T} } \right) $$

Here, the reward is represented as the weighted sum of two parts determined by parameter *λ*: (1) the reward *R*_*c*_ was provided by the prediction model, and (2) the reward *R*_*d*_ was calculated by discriminator neural network *D*, which was trained with generator simultaneously by minimizing the following loss function:

$$ L\left( \theta \right) = \mathop \sum \limits_{y \in Real} \left( {logD\left( {y_{1:T} } \right)} \right) + \mathop \sum \limits_{y \in Fake} \left( {log\left( {1 - D\left( {y_{1:T} } \right)} \right)} \right) $$

With the policy gradient optimization, the final model generated many different desired molecules which were predicted as active ligand against a given target and were similar to the chemical compounds in the ligands set. In the following section DrugEx and its performance is compared with these two methods.

The code of REINVENT and ORGANIC was downloaded from GitHub and executed with default parameters (*σ* = 60 in REINVENT and *λ* = 0.5 in ORGANIC). The prior network in REINVENT and generative network in ORGANIC were initialized with the pre-trained model, and the agent network in REINVENT was initialized with the fine-tuned model to make sure it could also employ this information. The RF-based predictor with ECFP6 was exploited as reward function for both methods identical to our own implementation. After these models were trained, 10,000 SMILES sequences were generated for performance comparison with each other (Table 1). Our method generated molecules that had the larger diversity at *ε* = 0.1. While DrugEx did not outperform REINVENT based on the percentage of unique desired SMILES, this value was improved dramatically and closely resembled that of REINVENT at *ε* = 0.01. In addition, although most of the molecules generated by these methods were drug-like molecules (Fig. 10), we found that molecules generated by our method covered the whole region of chemical space occupied by known active ligands. Conversely, molecules generated by both REINVENT and ORGANIC only covered a small fraction of the desired chemical space and were mostly centered in Rule-of-5 compliant chemical space even though the chemical space for the A_2A_R transcends this region of space. To further compare the chemical space occupied by the molecules generated by the different methods, the k-means algorithm was employed to cluster the active ligands in the *A2AR* set and generated molecules into 20 clusters with the ECFP6 fingerprints of (a) the full compound structure, (b) the Murcko scaffold and, (c) the topological Murcko scaffold (Additional file 1: Figure S4). The results indicated that the generated molecules by DrugEx covered all clusters that contain active ligands in the *A2AR* set, while some of these clusters were not covered by REINVENT and ORGANIC. Furthermore, the distribution of the molecules in each cluster generated by DrugEx more closely resembled the distribution by the active ligands in the *A2AR* set than was the case with either REINVENT or ORGANIC.

Previous work on the binding mechanism between the A_2A_R and its ligands identified a number of critical substructures that play an important role to improve binding affinity [39]. For example, the oxygen in the furan ring of ZM241385 and related ligands can form a hydrogen bond with residue N253, the purine ring acts as hydrogen bond donor to N253 and forms π-π interaction with F168 [7]. However, molecules containing such a furan ring tend to be blocking the receptor (antagonists) rather than activating it (agonists). Hence, while the furan ring is common in the set of known A_2A_R ligands, its presence might not always be favorable for generated ligands. Moreover, fused rings have been shown in general to be important in the chemical structure of drugs [40]. Therefore, we compared the percentage of molecules containing furan rings, fused rings, and benzene rings. Only 0.20% of the desired molecules generated by REINVENT contained a fused ring (Table 2) while they were present in 79.09% of active ligands in the *A2AR* set. Similarly, ORGANIC only generated a very low percentage of molecules containing a fused ring system (0.02%).

**Table 2:** Comparison of the percentage of important substructures contained in the molecules generated by the different methods and the molecules in the *ZINC* and *A2AR* sets

	Fused ring (%)	Furan ring (%)	Benzene ring (%)
DrugEx (Pre-trained)	9.12	82.32	61.48
DrugEx (Fine-tuned)	60.69	66.35	65.62
REINVENT	0.20	95.26	61.98
ORGANIC	0.02	99.96	39.45
Pre-trained	24.22	4.51	63.31
Fine-tuned	76.33	23.82	72.85
*ZINC*	26.66	3.86	63.97
*A2AR*			
Active	79.09	40.29	75.33
Inactive	76.73	9.33	70.88

These methods contained DrugEx with pre-trained and fine-tuned model as different *G*_*φ*_ (in the parentheses), REINVENT, ORGANIC, Pre-trained model, and Fine-tuned model

With the pre-trained network as *G*_*φ*_, DrugEx produced 9.12% of molecules containing fused rings, while the fine-tuned network improved the percentage of molecules containing fused rings up to 60.69%. For furan rings a similar image arises, 95.26% and 99.96% of molecules generated by REINVENT and ORGANIC contained a furan ring, respectively, while this percentage was only 40.29% for known active ligands. By comparison, in DrugEx, 82.32% of molecules contained a furan ring under the pre-trained network as *G*_*φ*_, similar to the other two methods. However, when the fine-tuned network was used this rate decreased substantially to 66.35%.

**Fig. 11:**
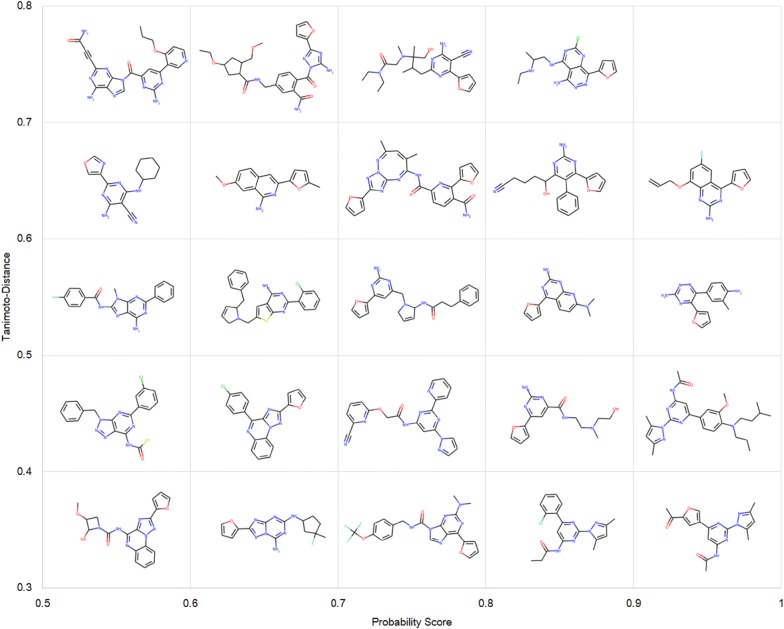
24 Candidate molecules were selected from 10,000 SMILES sequences generated by DrugEx. These molecules were ordered by the probability score given by the predictor and Tanimoto-distance to *A2AR* set

REINVENT and ORGANIC have been reported to generate various molecules containing different fused ring structures against DRD2 [21, 25]. One possible reason they were not able to do so here might lie in the bias of *A2AR* set. In Table 2, we noticed that there were more active ligands containing a furan ring than inactive ligands (fourfold difference). This led to both methods only generating molecules containing a furan ring which were prone to be predicted as active. However, both methods neglected to construct more complicated fused rings which is a decisive difference between active and inactive ligands in the *A2AR* set. These results indicate that DrugEx is more robust to overcome the bias of the training set to generate more similar compounds to known A_2A_R ligands (tuned for the target chemical space) and less generic SMILES sequences. Hence, we consider these molecules more appropriate drug candidates against A_2A_R than the molecules produced by REINVENT and ORGANIC. As an example, 24 candidate molecules generated by DrugEx were selected and are shown in Fig. 11 ordered by the probability score and Tanimoto-distance to the *A2AR* set.

In REINVENT, the pre-trained model acted as “priori” in the Bayesian formula to ensure that the generated SMILES are drug-like molecules. The final model was trained by improving the probability of desired generated SMILES while maintaining the probability of undesired generated SMILES similar to the pre-trained model. In DrugEx the pre-trained model was *only* used for initialization and did not directly affect the training process and performance evaluation. The mechanism of DrugEx appears quite similar to a genetic algorithm (GA) previously developed in our group for de novo drug design [41]. The exploration strategy can be regarded as “random mutation” in a GA context for sequence generation. Instead of changing the token selection directly, this manipulation just changed the probability distribution of each token in the vocabulary. Furthermore, although “crossover” manipulation was not implemented here, such mutations can still help the model search the unfamiliar chemical space in which the molecules do not have a high probability to be sampled. In contrast to ORGANIC, there was no need to construct another neural network specifically to measure the similarity between generated and real molecules, saving valuable time and resources required to train and select appropriate parameters. Hence, we conclude that molecules generated by DrugEx can be regarded as reasonable drug candidates for A_2A_R.

## Conclusion and future prospects

In this study a new method is proposed to improve the performance of deep reinforcement learning to generate SMILES based ligands for targets of interest. Applied to the A_2A_R, generated molecules had high diversity combined with chemical and predicted biological properties similar to known active compounds. Previous work has shown that RL cannot guarantee the model to generate molecules distributed over chemical space comparable to ligands of a target of interest. To solve this problem, another well-trained RNN model was employed as exploration strategy to force the model to enlarge the chemical space of the generated molecules during the training process of RL. Compared with other DL-based methods, DrugEx generated molecules with larger chemical diversity while maintaining a higher average similarity to known active ligands. However, the tradeoff is that slightly more inactive or duplicated molecules are being generated.

In future work, our aim is to update DrugEx with multi-objective optimization for polypharmacology. As a given drug (candidate) likely binds to unexpected targets (i.e. off-target efficacy) which can cause side-effects [42]. Incorporating multiple objectives in SMILES generation will allow the search for ways to eliminate potential off-target affinity.

## Additional file

**Additional file 1: Table S1.** All tokens in vocabulary for SMILES sequence construction with RNN model. **Figure S2.** The chemical space of generated molecules by pre-trained models, traditional reinforced model and active ligands in the A2AR set. **Figure S3.** The performance of DrugEx with different *G*_*φ*_ (pre-trained and fine-tuned model) and hyperparameters (including *ε* and *β*). **Figure S4.** The percentage of molecules in 20 groups clustered by k-means algorithm on ECFP6 fingerprints of generated molecules with full compound (A), Murcko scaffold (B) and topological Murcko scaffold (C).

## Data Availability

The data used in this study is publicly available ChEMBL data, the algorithm published in this manuscript is made available via GitHub, https://github.com/XuhanLiu/DrugEx.

## Abbreviations

A_2A_R: adenosine A_2A_ receptor
AUC: Area under the curve
DL: deep learning
DNN: Deep Neural Network
DRD2: dopamine D2 receptor
ECFP: Extended Connectivity Fingerprint
GA: genetic algorithm
GAN: generative adversarial network
GPCR: G Protein-Coupled Receptors
GRU: gated recurrent unit
MW: molecular weight
NB: Naïve Bayesian
PCA: principal component analysis
PhysChem: physicochemical descriptors
QSAR: quantitative structure-activity relationship
RBF: radial basis function
ReLU: rectified linear unit
RF: Random Forest
RL: reinforcement learning
RNN: recurrent neural network
ROC: receiver operator characteristic
SVM: Support Vector Machine
t-SNE: t-distributed stochastic neighbor embedding
