# Supplementary material for: An exploration strategy improves the diversity of de novo ligands using deep reinforcement learning: a case for the adenosine A2A receptor
Source: J Cheminform. 2019 May 24;11:35. doi: 10.1186/s13321-019-0355-6 (PMC6534880; doi:10.1186/s13321-019-0355-6)
Supplement: Supplementary file 1 — Additional file 1: Table S1. All tokens in vocabulary for SMILES sequence construction with RNN model. Figure S2. The chemical space of generated molecules by pre-trained models, traditional reinforced model and active ligands in the A2AR set. Figure S3. The performance of DrugEx with different Gφ (pre-trained and fine-tuned model) and hyperparameters (including ε and β). Figure S4. The percentage of molecules in 20 groups clustered by k-means algorithm on ECFP6 fingerprints of generated molecules with full compound (A), Murcko scaffold (B) and topological Murcko scaffold (C). [file 13321_2019_355_MOESM1_ESM.docx]

**Additional file 1**

***An Exploration Strategy Improves the Diversity of de novo Ligands Using Deep Reinforcement Learning – A Case for the Adenosine A_2A_ Receptor***

Xuhan Liu^1^, Kai Ye^2^, Herman W. T. van Vlijmen^1,3^, Adriaan P. IJzerman^1^, Gerard J. P. van Westen^1, *^

^1^Drug Discovery and Safety, Leiden Academic Centre for Drug Research, Einsteinweg 55, Leiden, The Netherlands

^2^Omics and Omics informatics, Xi’an Jiaotong University, 28 Xianning W Rd, Xi’an, China

^3^Janssen Pharmaceutica NV, Turnhoutseweg 30, B-2340, Beerse, Belgium

**^*^**To whom correspondence should be addressed: Gerard J. P. van Westen, Drug Discovery and Safety, Leiden Academic Centre for Drug Research, Einsteinweg 55, Leiden, The Netherlands. Tel: +31-71-527-3511. Email: [gerard@lacdr.leidenuniv.nl](mailto:gerard@lacdr.leidenuniv.nl).

Email Address of other authors: (1) Xuhan Liu: [x.liu@lacdr.leidenuniv.nl](mailto:x.liu@lacdr.leidenuniv.nl); (2) Kai Ye: [kaiye@xjtu.edu.cn](mailto:kaiye@xjtu.edu.cn); (3) Herman W. T. van Vlijmen: [hvvlijme@its.jnj.com](mailto:hvvlijme@its.jnj.com); (4) Adriaan P. IJzerman: [ijzerman@lacdr.leidenuniv.nl](mailto:ijzerman@lacdr.leidenuniv.nl).

**Table S1: All tokens in vocabulary for SMILES sequence construction with RNN model.**

| **Atoms** | | | | **Bonds** | | | **Controls** | | |
| --- | --- | --- | --- | --- | --- | --- | --- | --- | --- |
| **Common**  **Atoms** | | | **Aromatic Atoms** | | **--** | **Rings** | | **Branchs** | **On-Off** |
| B  C  F  I  Cl  N  O  P  Br  S | [B-]  [BH-]  [C+]  [C-]  [CH-]  [CH]  [C]  [N+]  [NH+]  [N] | [O-]  [O]  [P+]  [PH]  [Br+]  [S+]  [SH]  [SiH3]  [SiH]  [Si]  [Sn] | [cH-]  [n+]  [nH]  [s+]  c  n  o  p  s | | -  =  # | 1  2  3  4  5  6  7  8  9 | | (  ) | GO  EOS |

Considering that there are no drug-like molecules containing more than 10 rings, we omitted the token “0” and “%” for the construction of more than 10 rings. In addition, we ignored the isomerism of molecules and ionic bond, therefore we removed the “@”, ‘\’, ‘/’, ‘.’ and all metal ion.


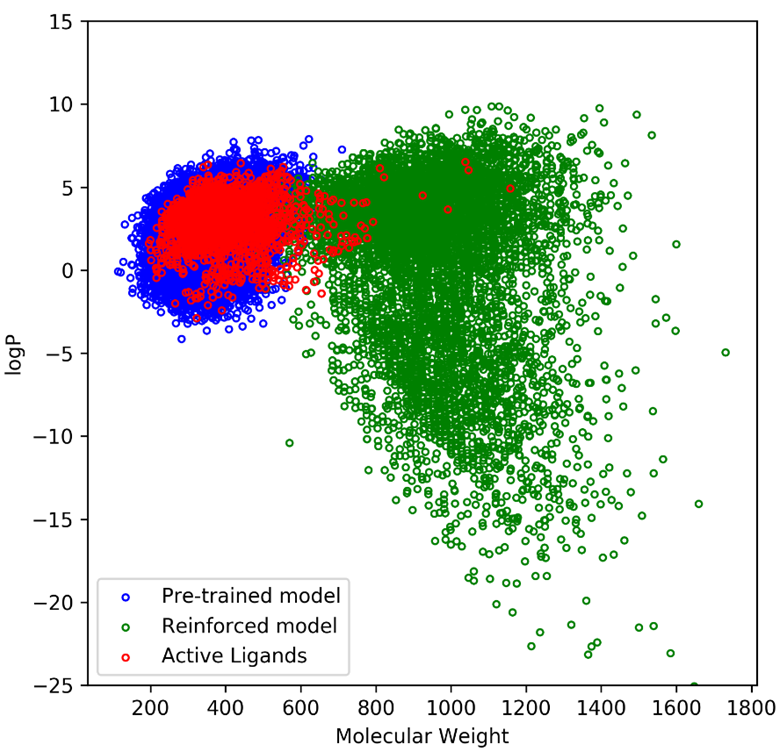


**Figure S2: The chemical space of generated molecules by pre-trained models, traditional reinforced model and active ligands in the *A2AR* set.** The chemical space was represented as logP ~ MW. The generated molecules by pre-trained model covered the greater part of space of known active ligands, while the molecules generated by reinforced model were distributed in a distinct region, which cannot be regarded drug-like although the compounds were predicted as active ligands.

**
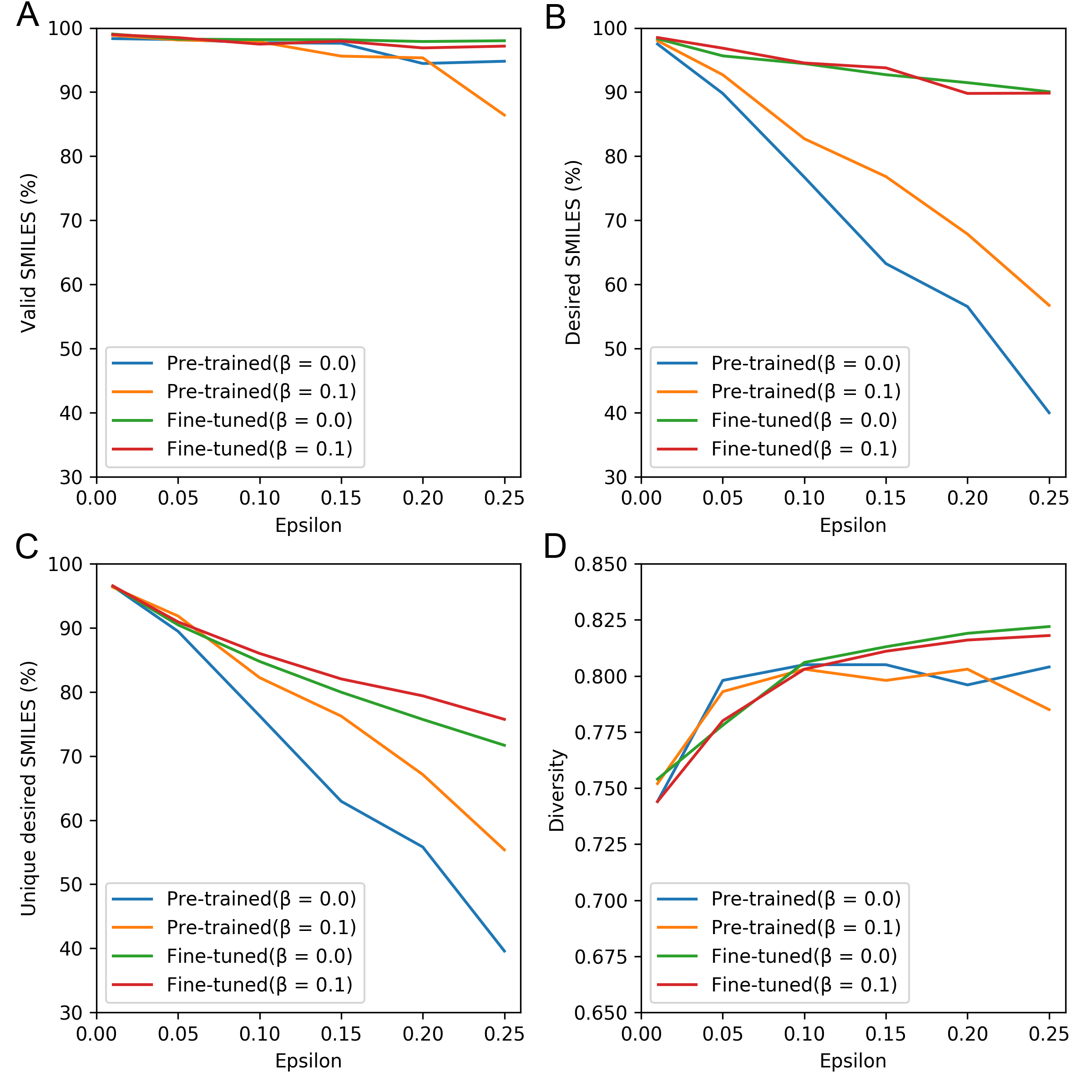
**

**Figure S3: The performance of DrugEx with different *G_φ_* (pre-trained and fine-tuned model) and hyperparameters (including *ε* and *β).*** These performances included the percentage of valid SMILES (A), desired SMILES (B) and unique desired SMIIES (C) and diversity (D).

**
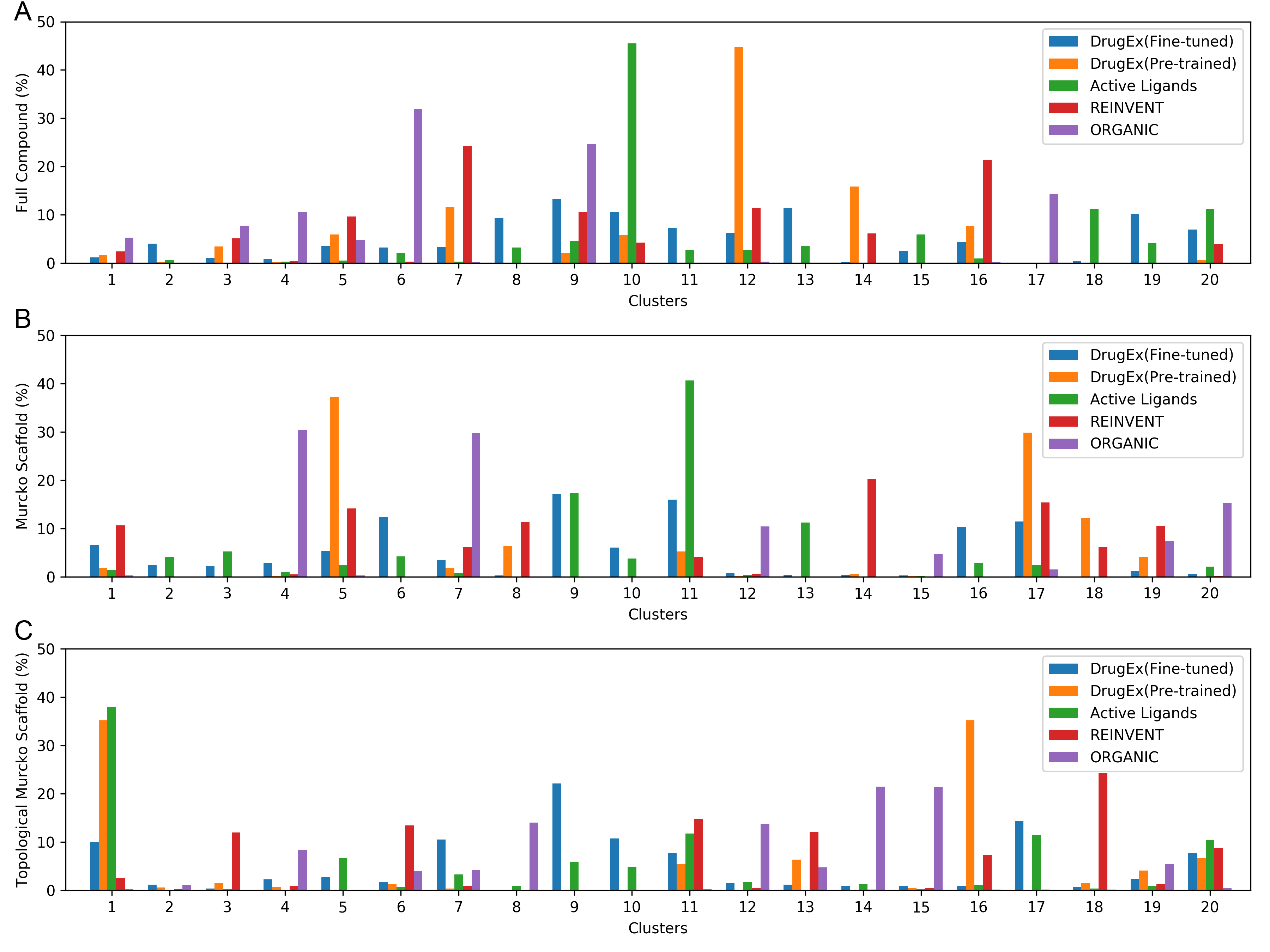
**

**Figure S4: The percentage of molecules in 20 groups clustered by k-means algorithm on ECFP6 fingerprints of generated molecules with full compound (A), Murcko scaffold (B) and topological Murcko scaffold (C)*.*** These molecules included active ligands in *A2AR* dataset and molecules generated by REINVENT, ORGANIC and DrugEx with different *G_φ_* (shown in the parentheses)
